# The CIPRUS study, a nurse-led psychological treatment for patients with undifferentiated somatoform disorder in primary care: study protocol for a randomised controlled trial

**DOI:** 10.1186/s13063-017-1951-2

**Published:** 2017-05-03

**Authors:** Kate Sitnikova, Stephanie S. Leone, Lyonne N. L. Zonneveld, Harm W. J. van Marwijk, Judith E. Bosmans, Johannes C. van der Wouden, Henriëtte E. van der Horst

**Affiliations:** 1 Department of General Practice and Elderly Care Medicine, Amsterdam Public Health Research Institute, VU University Medical Center, Van der Boechorststraat 7, 1081 BT Amsterdam, The Netherlands; 2 Department of Public Mental Health, Trimbos Institute: Netherlands Institute of Mental Health and Addiction, Da Costakade 45, 3521 VS Utrecht, The Netherlands; 3 Department of Anaesthesiology, Academic Medical Center, University of Amsterdam, Meibergdreef 9, 1105 AZ Amsterdam, The Netherlands; 4 Centre for Primary Care, Institute of Population Health, University of Manchester, Manchester, UK; 5 Department of Health Sciences, Amsterdam Public Health Research Institute, Faculty of Earth and Life Sciences, Vrije Universiteit Amsterdam, De Boelelaan 1105, 1081 HV Amsterdam, The Netherlands

**Keywords:** General practice, Primary care, Problem-solving treatment (PST), Undifferentiated somatoform disorder, Medically unexplained physical symptoms, Cost-effectiveness

## Abstract

**Background:**

Up to a third of patients presenting medically unexplained physical symptoms in primary care may have a somatoform disorder, of which undifferentiated somatoform disorder (USD) is the most common type. Psychological interventions can reduce symptoms associated with USD and improve functioning. Previous research has either been conducted in secondary care or interventions have been provided by general practitioners (GPs) or psychologists in primary care. As efficiency and cost-effectiveness are imperative in primary care, it is important to investigate whether nurse-led interventions are effective as well. The aim of this study is to examine the effectiveness and cost-effectiveness of a short cognitive behavioural therapy (CBT)-based treatment for patients with USD provided by mental health nurse practitioners (MHNPs), compared to usual care.

**Methods:**

In a cluster randomised controlled trial, 212 adult patients with USD will be assigned to the intervention or care as usual. The intervention group will be offered a short, individual CBT-based treatment by the MHNP in addition to usual GP care. The main goal of the intervention is that patients become less impaired by their physical symptoms and cope with symptoms in a more effective way. In six sessions patients will receive problem-solving treatment. The primary outcome is improvement in physical functioning, measured by the physical component summary score of the RAND-36. Secondary outcomes include health-related quality of life measured by the separate subscales of the RAND-36, somatization (PHQ-15) and symptoms of depression and anxiety (HADS). Problem-solving skills, health anxiety, illness perceptions, coping, mastery and working alliance will be assessed as potential mediators. Assessments will be done at 0, 2, 4, 8 and 12 months. An economic evaluation will be conducted from a societal perspective with quality of life as the primary outcome measure assessed by the EQ-5D-5L. Health care, patient and lost productivity costs will be assessed with the Tic-P.

**Discussion:**

We expect that the intervention will improve physical functioning and is cost-effective compared to usual care. If so, more patients might successfully be treated in general practice, decreasing the number of referrals to specialist care.

**Trial registration:**

Dutch Trial Registry, identifier: NTR4686, Registered on 14 July 2014.

**Electronic supplementary material:**

The online version of this article (doi:10.1186/s13063-017-1951-2) contains supplementary material, which is available to authorized users.

## Background

Physical symptoms that cannot be sufficiently explained by organic pathology are commonly presented by patients in all health care settings [1, 2]. In primary care up to one third of patients present these medically unexplained physical symptoms (MUPS) to the general practitioner (GP) [3]. Most of these symptoms are self-limiting, but some persist and cluster. Up to a third of patients presenting with such symptoms in primary care can be diagnosed with a somatoform disorder [3–5]. The most common type of somatoform disorder is undifferentiated somatoform disorder (USD), including patients who suffer from at least one impairing unexplained physical symptom lasting longer than 6 months [6]. USD is associated with considerable functional impairment and reduced quality of life, which in turn results in a high illness burden. Anxiety and depression are comorbid in at least 13.7% of the cases [7, 8] and may aggravate symptoms and functional limitations [5].

Additionally, USD is associated with high health care costs due to frequent and excessive health care use, repeated diagnostic procedures and high lost productivity costs [9, 10]. In 2010 the total costs of all somatoform disorders in Europe amounted to €21 billion, which was considered to be a conservative estimate [11, 12].

Previous research shows that only half of patients with USD seek help from a mental health care provider [13]. However, several reviews on treatment of MUPS and somatoform disorders show that cognitive behavioural therapy (CBT) is effective, with moderate effect sizes [14, 15]. Moreover, CBT interventions are effective in treating anxiety and depression [16] that often co-occur with USD.

A Cochrane review on non-pharmacological interventions for MUPS and somatoform disorders identified eight studies that recruited patients from primary care [14]. However, in only two of these studies treatment was actually performed in the primary care setting [17]. In one study [18], psychologists from secondary care performed a CBT-based group training within general practices. This training proved to be effective in increasing physical and emotional functioning and quality of life. Another study also offered a group intervention in general practice, but the treatment was given by specifically trained GPs and psychosomatic specialists [17]. This intervention was effective in improving mental but not physical functioning. However, the group formats may not appeal to all patients and take considerable time.

Less robust evidence shows that psychotropic medication, such as antidepressants, may also have some effect on the symptoms but these can induce dependence and may have side effects [19]. Also, a recent pilot study on a brief, multimodal psychosomatic therapy, combining physical and psychological components and delivered by physiotherapists showed improvement in perceived symptom severity, somatization and hyperventilation, but larger trials are needed to draw further conclusions [20].

Meanwhile, patients with USD frequently visit their GP [12]. However, to support and treat these patients can be a challenging task [21, 22]. GPs may feel powerless because they cannot find a somatic cause for the physical symptoms with which these patients typically present, and the patients themselves may fear serious disease [23]. In some cases, this may lead to unnecessary referrals to medical specialists for diagnostic and therapeutic purposes [24, 25]. Although GPs recognise the need to discuss psychological issues with these patients, this is often not feasible due to time constraints or GPs may feel ill-equipped to do so themselves [21]. Knowledge about treatment of USD in primary care and its cost-effectiveness in comparison with usual care is lacking.

Currently, the contribution of the mental health nurse practitioner (MHNP) within general practices in The Netherlands is increasing. A part of mental health care, in the form of short psychological treatment or coaching sessions, is taken over from the GP by the MHNP. MHNPs work within the general practice, and are trained to provide short-term psychological treatment. They are seemingly in a good position to offer psychological help to patients with USD in a more accessible way, provided such tasks are clear and there is evidence that such extra care is effective. However, they do not yet have a standard evidence-based protocol for treating these patients. We will, therefore, adapt an existing and effective secondary care protocol for primary care. To the best of our knowledge no previous research on the effectiveness of individual treatment conducted by primary care health care workers, such as MHNPs, has been executed yet.

The aim of this study is to examine the effectiveness and cost-effectiveness of a short CBT-based psychological treatment for patients with USD provided by MHNPs, in comparison with usual care.

## Methods/design

This protocol was developed in accordance with the Standard Protocol Items: Recommendations for Interventional Trials (SPIRIT) Statement. For the SPIRIT Figure see Additional file 1 and for the SPIRIT Checklist see Additional file 2. We will conduct a parallel-group, multicentre, cluster randomised controlled trial in 39 primary care centres (a list of the participating centres is provided in Additional file 3). Cluster randomisation will take place at the MHNP and general practice level in order to avoid contamination between the intervention and control conditions. Clusters will be composed by matching MHNPs to all participating general practices where the MHNP works and to all other MHNPs who also work in these general practices. The entire cluster consisting of one or more MHNPs and general practices will then be randomly assigned to the intervention or to care as usual (CAU) group, prior to the inclusion of patients. An independent statistician, not involved in the selection of the practices and MHNPs, will carry out the randomisation. In order to balance the size of the intervention and CAU groups, randomisation will be stratified according to cluster size (small: less than 5000 patients, and large: 5000 patients or more). Assessments will take place at baseline (T0), during the intervention period at approximately 2 months (T1), directly after the intervention period at 4 months (T2), at 8 months (T3) and at 12 months (T4) after baseline.

This study is registered at the Dutch Trial Registry (NTR4686) and was approved by the VU Medical Center Ethics Committee. It will be conducted according to the principles of the Declaration of Helsinki (version 2013). Important protocol modifications will be communicated with the Dutch Trial Registry and the VU Medical Center Ethics Committee.

### Participants

Participants will be recruited from various general practices and care groups situated in different geographical locations in The Netherlands. Patients will be eligible for the study if they are 18 years or older and meet the criteria for USD according to the *Diagnostic and Statistical Manual of Mental Disorders, version IV* (DSM-IV) [6].

Patients will be excluded from participation in the study if they have a medical or psychological disorder explaining their symptoms, a severe psychiatric disorder (e.g. psychotic disorders), are currently receiving psychological treatment for USD or have poor language skills or handicaps that prevent them from understanding the intervention. Patients can withdraw from the study at any time for any reason without any consequences.

### Inclusion procedure

The researchers and GPs will select adult patients (aged 18 years or older) from the GP’s electronic database, who consulted the GP with one or more symptoms from the Robbins list [26] (Table 1) at least twice in the previous 3 months. The presented symptom does not necessarily have to be the same for each visit. The Robbins list consists of 23 physical symptoms that are associated with functional somatic syndromes. The symptoms on this list are likely to be medically unexplained if they lack an accompanying ‘diagnostic’ or ‘disease’ International Classification of Primary Care (ICPC) code (i.e. ICPC code >70). Following this step, the participating GPs will check the selected patients for exclusion criteria in order to, for example, avoid inclusion of patients with actual somatic pathology.

**Table 1:** Symptoms from the Robbins list [26]

1. Back pain
2. Joint pain
3. Extremity pain
4. Headaches
5. Weakness
6. Fatigue
7. Sleep disturbance
8. Difficulty concentrating
9. Loss of appetite
10. Weight change
11. Restlessness
12. Thoughts slower
13. Chest pain
14. Shortness of breath
15. Palpitations
16. Dizziness
17. Lump in throat
18. Numbness
19. Nausea
20. Loose bowels
21. Gas or bloating
22. Constipation
23. Abdominal pain

**Fig. 1:**
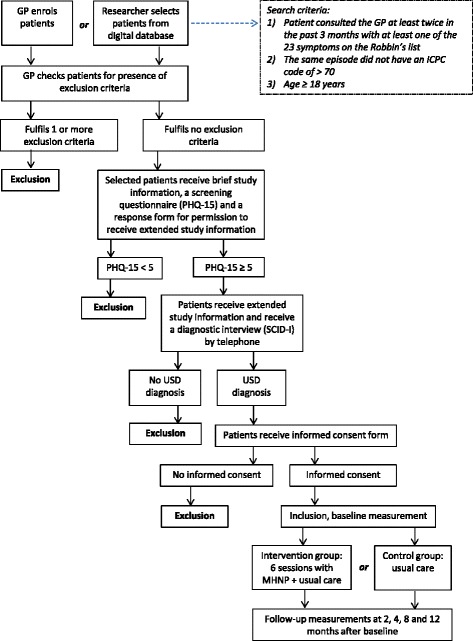
*GP g*eneral practitioner, *ICPC* International Classification of Primary Care, *PHQ-15* the Patient Health Questionnaire somatization scale, *SCID-I* Structured Clinical Interview for DSM-IV Axis I disorders, *USD* undifferentiated somatoform disorder, *MHNP* mental health nurse practitioner

Patients identified as potentially eligible will then receive brief information about the study and the Patient Health Questionnaire somatization scale (PHQ-15) [27] from their GP. Patients who are interested in participation in the study and who have a PHQ-15 score of 5 (low symptom severity) or higher will receive extensive study information. We chose the cut-off point for low symptom severity in order to make sure that patients with disabling somatic complaints are not wrongly excluded at this point. Patients will then be invited to participate in a clinical interview (Structured Clinical Interview for DSM-IV Axis I Disorders (SCID-I)) to assess DSM-IV criteria for USD. Trained members of the research team will administer the interview by telephone. Patients meeting the DSM-IV criteria for USD (the presence of one or more medically unexplained physical symptoms that last at least 6 months and significantly impair functioning/quality of life) will receive an Informed Consent Form. After completing the Informed Consent Form and sending it back to the researcher they will be included in the intervention or CAU group based on the allocation of the MHNP and general practice to which they belong. An overview of the inclusion procedure is provided in Fig. 1.

### The intervention

The intervention consists of extra care in addition to usual care. We realise that, specifically for this group, acknowledging and receiving help partly depends on the willingness to accept psychological assistance. Therefore, we hope that providing the intervention in their own general practice by their own MHNP will lower possible barriers to receiving psychological help.

The intervention consists of six individual sessions of 30 min each. The aim of the treatment is to improve physical functioning by helping patients cope with the consequences of their physical symptoms and with everyday problems in general. The rationale for the intervention is based on the consequences model for somatoform complaints [28, 29].

The consequences model for somatoform complaints has been found to be effective in previous Dutch randomised intervention studies [18, 30] and it is used in secondary mental health care. The model focusses on the consequences or problems that arise due to somatoform complaints rather than on the causes of somatoform complaints which are by definition unknown. In our study, we use the consequences model modified by Zonneveld et al. [31]. The modified consequences model assumes that physical symptoms lead to various consequences in the daily life of patients which are in fact survival strategies in reaction to the physical symptoms. Although these survival strategies must have been beneficial at the beginning (otherwise they would not have been developed) they can aggravate symptoms and become harmful or devastating in the long run. In the model, patients can improve (physical) functional and quality of life by enhancing the more beneficial survival strategies in the long run instead of the harmful ones. Identified consequences or problems are then tackled using cognitive behavioural problem-solving techniques which will be learned and applied by patients in a stepwise manner according to the steps outlined in problem-solving therapy (PST). The goal is to help patients develop more helpful survival strategies in the long run. PST has proven to be effective for depression in primary care and is suitable to be carried out by trained GPs or nurses [32, 33]. Although PST has been investigated less thoroughly in patients with USD, several studies show promising results [18, 34, 35]. Patients become more skilled in developing (helpful) survival strategies or solutions by working through the following seven steps: (1) identifying and specifying a problematic situation, (2) setting a clear goal for resolving this problematic situation, (3) formulating as many survival strategies or solutions as possible to reach this goal, (4) making a cost-benefit analysis for each strategy or solution, (5) selecting the most favourable strategy or solution, (6) specifying the necessary steps to implement the strategy or solution and (7) implementing the strategy or solution and evaluating its results. An intervention protocol was developed in which the intervention is described in detail. More information about the intervention can be requested from the first author.

### Training of mental health nurse practitioners

Before administering the intervention, all MHNPs in the intervention group will receive two group-training sessions of 3 to 3.5 h each, depending on the size of the group, during a 2-week period (one session a week). A registered clinical psychologist with broad clinical expertise in treating patients with somatoform complaints will lead the training sessions. The aim is to train the MHNPs in understanding and applying the intervention according to the intervention protocol. In the first training session the theoretical background of MUPS, USD and the modified consequences model will be given. In the second training session, the steps of PST will be introduced, explained, modelled by the clinical psychologist and finally practised by the MHNPs by applying the treatment to a co-trainee. In between the two training sessions, MHNPs will be asked to go through the PST steps to solve a minor problem of their own as homework. MHNPs will also receive a copy of the treatment protocol that will serve as a guideline for the intervention.

### Feasibility testing and ongoing supervision

In order to determine whether the intervention is feasible in practice, two MHNPs will be asked to pilot test the protocol with a patient. Also, two patients will be asked about their opinion on the treatment protocol. Their feedback will be collected and the protocol will be adjusted if needed. During the intervention period a supervision session with the clinical psychologist (by telephone or face-to-face) will be offered. MHNPs can also contact the researcher during the entire intervention period for any questions regarding the treatment and the study.

Furthermore, five randomly selected MHNPs will be asked to record all sessions with all of their patients. The researcher (KS) will listen to the recordings and identify topics in the obstacles encountered by the MHNPs. The researcher will communicate this with the clinical psychologist, so that these topics can be addressed during supervision.

### Care as usual (CAU) group

Patients in the CAU group will not be offered any additional specific intervention other than the care that they would usually receive from the GP and/or MHNP, carried out according to the guideline for medically unexplained symptoms by the Dutch College of General Practitioners (NHG) [36].

### Outcome measures

An overview of all outcome measures and the time points of assessments can be found in Table 2.

**Table 2:** Overview of assessment moments and outcome measurements

	Instrument	Baseline (T0)	2 months (T1)	4 months (T2)	8 months (T3)	12 months (T4)
Primary outcomes						
Physical functioning	PCS of RAND-36	x	x	x		x
Direct and indirect costs	Tic-P	x		x	x	x
Quality of life	EQ-5D-5L	x	x	x	x	x
Secondary outcomes						
HRQoL	RAND-36 subscales	x	x	x		x
Anxiety and depression	HADS	x	x	x		x
Number/severity of symptoms	PHQ-15	x	x	x		x
Potential mediators						
Health anxiety	Whitely Index	x	x	x		
Illness perceptions	Brief IPQ	x	x	x		
Cognitions and coping	CBRQ	x	x	x		
Social problem-solving skills	SPSI-R:S	x	x	x		
Mastery	Pearlin Mastery Scale	x	x	x		
Working alliance	WAI-SF^a^		x	x		

*PCS* physical component summary score, *RAND-36* RAND-36-item Health Survey, *Tic-P* Trimbos and iMTA questionnaire on Costs associated with Psychiatric Illness, *EQ-5D-5L* EuroQol 5D – 5 level version, *HRQoL* health-related quality of life, *HADS* Hospital Anxiety and Depression Scale*, PHQ-15* Patient Health Questionnaire somatization scale, *IPQ* Illness Perception Questionnaire, *CBRQ* Cognitive and Behavioural Responses Questionnaire, *SPSI-R:S* Social Problem-solving Inventory Revised: Short Form, *WAI-SF* Working Alliance Inventory-Short Form

^a^only administered in the intervention group

#### Primary outcomes

The primary clinical outcome of this study is the improvement in physical functioning during the total follow-up period measured by the physical component summary score (PCS) of the RAND-36-item Health Survey (RAND-36). The RAND-36 is a widely used, valid and reliable health-related quality of life (HRQoL) instrument comprising 36 items that assess eight health domains: physical functioning, role limitations caused by physical health problems, role limitations caused by emotional problems, social functioning, emotional wellbeing, energy/fatigue, pain and general health perceptions [37–39]. Physical and mental health summary scores (PCS and MCS, respectively) can be derived from the eight scales. The raw scores are transformed into scores ranging from 0 to 100 with a higher score indicating better functioning.

The primary outcome for the economic evaluation is quality of life as measured by the EuroQol 5D – 5 level version (EQ-5D-5L) [40]. The EQ-5D-5L is a standardised instrument including five health domains (mobility, self-care, usual activities, pain/discomfort and anxiety/depression). It comprises five items, one single item referring to each domain, that are assessed on a 5-point scale: 0 = ‘no problems with’, 1 = ‘slight problems with’, 2 = ‘moderate problems with’, 3 = ‘severe problems with’ and 4 = ‘unable to’. The health state indicated by patients on the EQ-5D-5L will be converted to a utility score using the Dutch EQ-5D-5L tariff [41]. The EQ-5D-5L utility scores at different time points will be used to calculate quality-adjusted life years (QALYs) using the area under the curve method. Changes between health states at different time points are considered to be linear. Also, the patient is asked to rate their general health on a 0–100 scale [40].

Societal costs will be assessed with the Trimbos and iMTA questionnaire on Costs associated with Psychiatric Illness (Tic-P) [42]. The Tic-P is an instrument that assesses self-reported health care utilisation, medication use, informal care, absenteeism from paid and unpaid work, and presenteeism. The costs of the intervention will be estimated using a bottom-up approach. For the valuation of health care utilisation and informal care, standard prices published in the Dutch costing guidelines will be used [42]. Medication use will be valued using prices of the Royal Dutch Society for Pharmacy (Z-index). The friction cost approach will be used to estimate absenteeism from paid work based on sex-specific mean wages in the Dutch population.

#### Secondary outcomes

Secondary outcome measures are the eight separate subscales scales of the RAND-36 (physical functioning, role limitations caused by physical health problems, role limitations caused by emotional problems, social functioning, emotional wellbeing, energy/fatigue, pain and general health perceptions) [39], severity of somatization (Patient Health Questionnaire 15-item somatic symptom scale (PHQ-15) [27]) and depressive and anxiety symptoms (Hospital Anxiety and Depression Scale (HADS) [43]). PHQ-15 is a somatic symptom scale derived from the Patient Health Questionnaire (PHQ). It comprises 15 items, each of which can be scored from 0 = ‘not bothered at all’ to 2 = ‘bothered a lot’, which results in a total score ranging from 0 to 30. Higher scores indicate higher somatic symptom severity. Scores of 5, 10 and 15 represent cut-off points for low, medium and high somatic symptom severity, respectively [27] .

The HADS is a 14-item instrument assessing symptoms of anxiety (seven items) and depression (seven items). There are four answer categories that are given scores of 0–3, resulting in a total score ranging from 0 to 21 for each scale. Higher scores on the HADS indicate more severe symptoms of anxiety and depression. A score of 0–7 indicates no depressive or anxiety disorder. A score of 8–10 indicates a possible depressive or anxiety disorder, whereas a score of 11–21 indicates a probable depressive or anxiety disorder [43].

#### Patient and illness characteristics

In order to examine which patients are most likely to benefit from the intervention, we will assess various potential effect modifiers. The choice of these factors was based in part on the Dutch multidisciplinary guideline for MUPS and somatoform disorders which advocates the use of three patient profiles: mild, moderate and severe symptom profile [36]. Severity depends on factors such as duration, severity and number of symptoms and comorbidity. The potential effect modifiers selected in this study are:

- Demographic factors: age, gender and education (self-report)
- Illness duration (self-report)
- Severity of somatization (somatization scale of the PHQ-15 [27])
- Physical comorbidity (self-report)
- Psychiatric comorbidity: anxiety and depression (HADS [43])

#### Potential mediators

The intervention is expected to have positive effects on physical functioning and quality of life through developing problem-solving skills and increasing adequate coping. Moreover, health anxiety and dysfunctional somatic causal attributions are thought to be important aggravating factors of somatoform disorders which are reflected in the new DSM-5 criteria for somatic symptom disorder (formerly somatoform disorders) [44]. Therefore, the following factors will be assessed as potential mediators:

- Problem-solving skills (Social Problem-solving Inventory [45])
- Health anxiety (Whiteley Index [46])
- Illness perceptions (brief IPQ [47])
- Coping and beliefs about symptoms (CBRQ [48])
- Mastery (Pearlin Mastery Scale [49])
- Working alliance (WAI-SF [50])

The Social Problem-solving Inventory Revised: Short Form (SPSI-R:S) measures an individual’s problem-solving strengths and weaknesses. It assesses five dimensions: positive problem orientation, rational problem solving, negative problem orientation, impulsive/careless style and avoidance style. It consists of 25 items that can each be scored on a 5-point Likert scale (0 = ‘not at all true for me’ to 4 = ‘extremely true for me’). Higher scores indicate greater effective social problem-solving skills [45].

The Whiteley Index is a short instrument measuring health anxiety and is often used in investigating symptoms of hypochondria. It consists of 14 items with two answer categories ‘yes’ and ‘no’. A higher score indicates greater health anxiety [46].

The brief Illness Perception Questionnaire (IPQ) is based on the widely used IPQ-R. It is designed in order to rapidly assess the cognitive and emotional representations of illness. It comprises nine items in total. Five items assess cognitive illness representations: consequences, timeline, personal control, treatment control and identity. Two items assess emotional representations: concern and emotions. One item assesses illness comprehensibility. The last item is an open-ended question assessing causal representation [47].

The Cognitive and Behavioural Responses Questionnaire (CBRQ) measures patients’ cognitive and behavioural responses to their illness. This tool has been developed to measure specific cognitions and coping styles in patients with physical symptoms. It consists of 41 items that can be rated on a 5-point Likert scale ranging from ‘strongly disagree’ to ‘strongly agree’. These items add up to four cognitive subscales: catastrophising, damaging beliefs, embarrassment avoidance and symptom focussing; and two behavioural subscales: all-or-nothing behaviour and avoidance/resting behaviour [48].

The Pearlin Mastery Scale measures the level of perceived control, or mastery, over situations in one’s life. It comprises seven items that are scored on a 5-point Likert scale, ranging from ‘completely disagree’ to ‘completely agree’ [49].

The Working Alliance Inventory-Short Form (WAI-SF) is a shortened version of the Working Alliance Inventory (WAI). It is used to measure the therapeutic alliance in an ongoing client-therapist interaction. It comprises 12 items that are scored on a 5-point Likert scale, ranging from ‘never or rarely’ to ‘very often’ [50].

### Factors influencing implementation

#### Non-response and patient satisfaction

Patients who do not want to participate in the study will be asked for reasons for non-participation. Participating patients will receive questions about satisfaction with the content and relevance of the treatment at the 4-month follow-up. The questions will cover topics such as: patients’ expectations and needs prior to treatment; whether the treatment lived up to their expectations; whether patients considered themselves to be the target audience (suffering from USD); patients’ reasons for participating; whether the treatment helped in the short and the long term; satisfaction with the duration of the treatment and whether they would recommend the treatment to someone else in the future. The above questions will be assessed using a 5-point Likert scale ranging from 1 = ‘very satisfied’ to 5 = ‘very unsatisfied’. Also, reasons for dropout will be assessed.

#### Evaluation MHNPs

The MHNPs in the intervention group will receive a questionnaire to evaluate their opinion about the content and relevance of the treatment. The questions will cover topics such as whether MHNPs felt the treatment was effective for the patients; whether the intervention protocol was useful to the MHNPs and whether they followed the protocol as they were asked to do. Potential reasons for non-compliance will be explored. The questions on the opinions of the MHNPs will also be assessed on a 5-point Likert scale. Additionally, MHNPs will be interviewed by telephone or in person to gain insight into factors that they deem relevant for successful implementation of the intervention in the future and what possible barriers they identify. The interviews will be recorded and transcribed for analysis.

### Handling and storage of data and documents

Data will be collected and stored digitally using the web database NetQuestionnaires. This database treats data with strict confidence and does not disclose data to any third parties without permission of the user. NetQuestionnaires also takes safety measures for collecting, storing and processing data to prevent unauthorised access. In case people prefer a paper version of the questionnaires, this will be sent by regular mail. The completed paper questionnaires will be stored in a locked closet at the Department of General Practice and Elderly Care Medicine. When working with data, subjects will be assigned with a code. The code list will be safeguarded by the principal investigator. Only the principal investigator and research assistants will be able to access the source data. Data will be kept for 15 years.

### Power calculation/sample size calculation

We based the sample size calculation on an expected increase in the primary clinical outcome PCS of the RAND-36 during the total follow-up period (4 and 12 months after baseline). Based on previous findings, we aim to detect a clinically relevant effect size of 0.4. This effect size was previously shown to be feasible in a similar population [18]. The clinically relevant difference on the PCS ranges from 3 to 5 points [51]. Based on an estimate of the standard deviation of the PCS score of 10 [52], our effect size corresponds to a 4-point difference between the intervention group and the CAU group. We assume a two-sided significance level of 5% and a power of 80%. The ratio of the number of subjects in the compared groups is 1:1 and there are two measurements of follow-up for our primary outcome. Although we assume that the results will be similar on both repeated measurements, we opted for a more conservative correlation coefficient of 0.5. Using linear mixed models with these values leads to a sample size of *n* = 74 patients per condition.

However, since our study is a cluster randomised controlled trial, we applied an additional correction for the ‘design effect’ [53] with an expected average cluster size of four. As previous research found that 90% of intra-class correlation coefficients (ICCs) in primary care research are smaller than 0.055 [54], we chose an ICC of 0.05. After applying the correction, the sample size resulted in *n* = 85 patients (1.15 × 74) per condition. Taking a potential dropout rate of 20% into account, we aim to include 106 (85/0.8) patients in each condition.

### Statistical analyses

#### Primary outcomes

Differences in the change scores between the intervention group and the CAU group on the PCS of the RAND-36 will be analysed with linear mixed models according to the intention-to-treat principle as outlined in the Consolidated Standards of Interventional Trials (CONSORT) Statement with extension to cluster randomised trials [55]. This analysis technique allows for the clustering of patients within MHNPs and for dependence of observations within individuals over time.

#### Secondary outcomes

Differences in change scores between the intervention group and the CAU group on the eight separate scales of RAND-36, HADS and PHQ-15 (secondary outcome measures) will also be analysed with linear mixed models according to the intention-to-treat principle.

#### Economic evaluation

*Cost-effectiveness analysis (CEA):* both a cost-effectiveness and cost-utility analysis will be performed from a societal perspective. The time period of the economic evaluation will be 12 months; therefore, discounting is not necessary. Sensitivity analyses will be performed to assess the robustness of the results using different assumptions regarding costs and effects.

Societal costs will be related to the following effect measures in the economic evaluation:

1. Physical functioning as measured by the RAND-36
2. Quality-adjusted life years (QALYs) based on the Dutch tariff for the EuroQol (EQ-5D-5L) [41]
3. Severity of somatization (PHQ-15) and mental health (HADS)

The analysis will be done according to the intention-to-treat principle. Missing cost and effect data will be imputed using a multiple imputation technique. Incremental cost-effectiveness ratios (ICERs) will be calculated by dividing the difference in mean total costs between the groups, by the difference in mean effects between the groups. Bootstrapping with 5000 replications will be used to estimate 95% confidence intervals around cost differences and the uncertainty surrounding the ICERs. Rubin’s rules will be used to pool the results from the different multiply imputed datasets. Uncertainty surrounding the ICERs will be graphically presented on cost-effectiveness planes. Cost-effectiveness acceptability curves showing the probability that the intervention was cost-effective in comparison with usual care for a range of different ceiling ratios will also be estimated [56]. We will adjust for confounders and effect modifiers, when necessary*.*

*Budget impact analysis (BIA):* in the BIA, the cost-effectiveness of the short-term psychological intervention and usual care will be extrapolated using a simple Markov model over a period of 5 years based on the estimates obtained from the proposed study. Societal, government and insurer perspectives will be considered. Different scenarios will be evaluated including the following: (1) the intervention is not implemented, i.e. all patients receive usual care, (2) the intervention is offered to the whole patient population, (3) the intervention is implemented over a period of 4 years (25% of the patient population per year) and (4) the intervention is only offered to specific subgroups of the potential patient population. These subgroups will be defined based on the results of the study (e.g. subgroups that particularly benefitted from the intervention).

The total number of patients eligible for the intervention will be estimated based on Dutch incidence and prevalence rates of USD. Resource utilisation will be calculated by multiplying the number of eligible patients with the resource utilisation rates obtained from the cost-effectiveness analysis. Different prices will be used to value resource use depending on the perspective of the analysis: Dutch standard costs for the societal perspective, actual Dutch Healthcare Authority (NZA) tariffs for the government perspective, and average NZA tariffs for the insurer perspective. Both resource use and annual costs will be presented over a 5-year period for all perspectives. Aggregated and disaggregated (e.g. GP care, secondary care, and productivity losses) total costs per year will be presented for the different perspectives and scenarios.

*Analysis of patients most likely to benefit from the intervention*: if power allows, interaction terms between potential effect modifiers (e.g. severity, comorbidity, gender) and group allocation will be tested in mixed regression models. In case of insufficient power, only exploratory analyses will be done.

*Mediation analyses*: mediation analyses will be conducted to determine whether the intervention affected physical functioning through changes in problem-solving skills, health anxiety, illness perceptions, coping, mastery and/or working alliance. The Krull and MacKinnon method [57] will be used for this purpose.

*Factors influencing implementation*: data will be obtained on participation rate, satisfaction with the intervention and characteristics of non-responders. Data collected from non-responders will be limited to demographic characteristics, such as age and gender and reasons for not participating in the study. Impeding and facilitating factors for implementation as observed by MHNPs will be ascertained by conducting interviews with the latter. The interviews will be transcribed verbatim. The transcripts will be analysed by coding the texts using Atlas.ti and themes will be identified and described.

## Discussion

To date, this is the first study to evaluate the effectiveness and cost-effectiveness of a short CBT-based treatment provided by MHNPs for patients with undifferentiated somatoform disorder in primary care versus usual care. The aim of the treatment is to improve physical functioning by increasing problem-solving skills to cope with consequences of the physical symptoms and with everyday problems in general. We assume that higher physical functioning and quality of life will result in lower health care-related and work-related costs.

A strength of this study is that the intervention is provided within the patients’ own general practices. Given the prevalence of USD and the large societal costs that accompany this disorder, there is an urgent need to provide easily accessible and affordable treatment for patients with USD. A previous study showed that providing psychological help in general practice was effective in improving quality of life [18]. However, the intervention in this study was conducted in a group setting by psychologists from secondary care who offered treatment to patients in general practice. This might not always be feasible and time-efficient. MHNPs, working in general practices and trained in providing (short) psychological treatment, are in a much more convenient position to help patients.

Furthermore, receiving psychological treatment in the patients’ general practice may create a safe and low-threshold environment for patients with USD, especially those who would otherwise not seek psychological help in the mental health care setting. Also, by focussing on the consequences and not on the causes of physical symptoms, the possible struggle about the cause of the symptoms is avoided. Regardless of the cause, patients suffer from consequences of USD. This approach may create more acceptance from the patients.

By testing our intervention directly within general practices and with MHNPs who are already employed there, this study has a high clinical relevance. If successful, the intervention is likely to be easily implemented in daily practice as the number of MHNPs employed in the general practices is growing. This is especially relevant in The Netherlands, as more emphasis is being placed on provision of mental health care services in general practice as a result of organisational changes in health care services.

Additionally, the current intervention combines a cognitive behavioural theoretical framework with PST intervention techniques for somatoform disorders. To date, PST has been widely investigated in depression [32, 58, 59] but rarely in somatoform disorders. One preliminary study containing 11 subjects who received PST found that PST was acceptable to patients and reduced symptoms, hypochondriacal preoccupation and psychiatric morbidity [35]. Another study investigated PST in 162 patients and found a positive impact on symptoms, functioning and costs, but in this study PST was combined with other CBT techniques [18].

A possible limitation to our study is that we use the diagnosis undifferentiated somatoform disorder (USD) as defined by the DSM-IV. The fifth edition of the DSM (DSM-5) has a new classification for the previous category somatoform disorders. Previously, the category of somatoform disorders included somatization disorder, undifferentiated somatoform disorder, conversion disorder, pain disorder, hypochondriasis, body dysmorphic disorder and somatoform disorder not otherwise specified. All of these somatoform disorders except hypochondriasis, body dysmorphic disorder and conversion disorder have now been categorised under somatic symptom disorder (SSD). Furthermore, for the classification of SSD the somatic symptoms do not have to be medically unexplained. Our study was designed and funded before DSM-5 was introduced. SSD is a new DSM-5 classification and issues such as its usefulness and accuracy are currently a topic of debate in the field in addition to proposals for modifications of the criteria for SSD [60–62]. Moreover, since its introduction, the DSM-5 has not yet been widely used in research and practice, and no appropriate diagnostic instrument equivalent to the SCID or the Mini International Neuropsychiatric Interview (MINI) was available at the time of recruitment of patients. Therefore, for practical reasons it was impossible to diagnose patients reliably and validly according to the DSM-5. Recently the Health Preoccupation Diagnostic Interview (HPDI), a new structured diagnostic interview for the classification of somatic symptom disorder and illness anxiety disorder, has been developed by Axelsson and colleagues [63]. However, this diagnostic interview has not yet been validated.

Another limitation is that, due to the nature of the intervention, it is not possible to blind patients, health care providers and researchers to treatment allocation.

A final point of consideration concerns the primary outcome measure, the physical component summary score (PCS) of the RAND-36. The PCS is a general physical functioning scale, comprising subscales measuring physical aspects of health. Because the PCS is a summary score, the total score may be somewhat insensitive to change, as potential changes on the separate subscales may not lead to a difference in the total score. This may make it more difficult to detect an effect on the PCS. However, several previous studies have successfully used the PCS scale as a primary or secondary outcome and were able to detect change [18, 64–67]. Despite potential shortcomings, we also chose the PCS as the primary outcome measure, because a more specific measure, such as the subscale ‘physical pain’ of the RAND-36, may focus on only a part of physical functioning, whereas we aim to investigate physical functioning in a more generic manner. After all, the aim of the intervention is not to reduce the symptoms, but to improve physical functioning as a whole. We will also separately investigate the changes on the separate subscales as a secondary outcome in order to see whether more specific changes take place.

Overall, if this study shows that the treatment is effective and cost-effective, the treatment could result in great benefits in primary care by making psychological treatments more available to patients with USD and providing broader treatment possibilities for primary care professionals. Quality of life of patients with USD may be improved and health care costs may be reduced.

### Trial status

The collection of data started in November 2015. Study results are expected to be available in April 2018. The recruitment of participants was ongoing at the time of manuscript submission.

## Additional files

Additional file 1: Schedule of enrolment, intervention and assessments. (DOC 39 kb)

Additional file 2: SPIRIT Checklist. (DOC 120 kb)

Additional file 3: List of participating primary care centres. (DOCX 15 kb)

## Abbreviations

BIA: Budget impact analysis
CAU: Care as usual
CBT: Cognitive behavioural therapy
CBRQ: Cognitive and Behavioural Responses Questionnaire
CEA: Cost-effectiveness analysis
DSM-IV (or DSM-5): *Diagnostic and Statistical Manual of Mental Disorders-IV* (or *-5*)
EQ-5D-5L: EuroQol 5D – 5 level version
GP: General practitioner
HADS: Hospital Anxiety and Depression Scale
HPDI: Health Preoccupation Diagnostic Interview
HRQoL: Health-related quality of life
ICC: Intra-class correlation coefficient
ICERs: Incremental cost-effectiveness ratios
ICPC: International Classification of Primary Care
IPQ: Illness Perception Questionnaire
MHNP: Mental health care practitioner
MINI: Mini International Neuropsychiatric Interview
NHG: The Dutch College of General Practitioners (Dutch: Nederlands Huisartsen Genootschap)
NZA: the Dutch Healthcare Authority (Dutch: Nederlandse Zorgautoriteit)
PCS: Physical component score
PHQ-15: Patient Health Questionnaire somatization scale
PST: Problem-solving treatment
QALYs: Quality-adjusted life years
RAND-36: RAND-36-item Health Survey
SCID: Structured Clinical Interview for DSM-IV
SSD: Somatic symptom disorder
SPSI-R:S: Social Problem-solving Inventory Revised: Short Form
Tic-P: Trimbos and iMTA questionnaire on Costs associated with Psychiatric Illness
USD: Undifferentiated somatoform disorder
WAI-SF: Working Alliance Inventory-Short Form

## References

[1] Henningsen P, Zipfel S, Herzog W (2007). Management of functional somatic syndromes. Lancet.

[2] Burton C (2003). Beyond somatisation: a review of the understanding and treatment of medically unexplained physical symptoms (MUPS). Br J Gen Pract.

[3] Steinbrecher N, Koerber S, Frieser D, Hiller W (2011). The prevalence of medically unexplained symptoms in primary care. Psychosomatics..

[4] Haller H, Cramer H, Lauche R, Dobos G (2015). Somatoform disorders and medically unexplained symptoms in primary care. Dtsch Arztebl Int..

[5] De Waal M, Arnold I, Eekhof J, van Hemert A (2004). Somatoform disorders in general practice: prevalence, functional impairment and comorbidity with anxiety and depressive disorders. Br J Psychiatry.

[6] American Psychiatric Association. Diagnostic and Statistical Manual of Mental Disorders. 4th ed. Washington, DC: American Psychiatric Association; 2000.

[7] Mergl R, Seidscheck I, Allgaier A, Möller H, Hegerl U, Henkel V (2007). Depressive, anxiety, and somatoform disorders in primary care: prevalence and recognition. Depress Anxiety.

[8] Janssens KAM, Zijlema WL, Joustra ML, Rosmalen JGM (2015). Mood and anxiety disorders in chronic fatigue syndrome, fibromyalgia, and irritable bowel syndrome: results from the LifeLines cohort study. Psychosom Med..

[9] Konnopka A, Schaefert R, Heinrich S, Kaufmann C, Luppa M, Herzog W (2012). Economics of medically unexplained symptoms: a systematic review of the literature. Psychother Psychosom.

[10] Barsky AJ, Orav EJ, Bates DW (2005). Somatization increases medical utilization and costs independent of psychiatric and medical comorbidity. Arch Gen Psychiatry.

[11] Bermingham SL, Cohen A, Hague J, Parsonage M (2010). The cost of somatisation among the working-age population in England for the year 2008–2009. Ment Health Fam Med.

[12] Gustavsson A, Svensson M, Jacobi F, Allgulander C, Alonso J, Beghi E (2012). Cost of disorders of the brain in Europe 2010. Eur Neuropsychopharmacol.

[13] Mack S, Jacobi F, Gerschler A, Strehle J, Höfler M, Busch MA (2014). Self-reported utilization of mental health services in the adult German population—evidence for unmet needs? Results of the DEGS1-Mental Health Module (DEGS1-MH). Int J Methods Psychiatr Res.

[14] Van Dessel N, Den Boeft M, Van der Wouden J, Kleinstäuber M, Leone S, Terluin B (2014). Non-pharmacological interventions for somatoform disorders and medically-unexplained physical symptoms (MUPS) in adults. Cochrane Database Syst Rev..

[15] Kroenke K, Swindle R (2000). Cognitive-behavioral therapy for somatization and symptom syndromes: a critical review of controlled clinical trials. Psychother Psychosom..

[16] Watts SE, Turnell A, Kladnitski N, Newby JM, Andrews G (2015). Treatment-as-usual (TAU) is anything but usual: a meta-analysis of CBT versus TAU for anxiety and depression. J Affect Disord..

[17] Schaefert R, Kaufmann C, Wild B, Schellberg D, Boelter R, Faber R (2013). Specific collaborative group intervention for patients with medically unexplained symptoms in general practice: a cluster randomized controlled trial. Psychother Psychosom.

[18] Zonneveld LNL, van Rood YR, Timman R, Kooiman CG, Van’t Spijker A, Busschbach JJV (2012). Effective group training for patients with unexplained physical symptoms: a randomized controlled trial with a non-randomized one-year follow-up. PLoS One.

[19] Kleinstäuber M, Witthöft M, Steffanowski A, van Marwijk H, Hiller W, Lambert MJ (2014). Pharmacological interventions for somatoform disorders in adults. Cochrane Database Syst Rev.

[20] Wortman MSH, Lucassen PLBJ, van Ravesteijn HJ, Bor H, Assendelt P, Lucas C (2016). Brief multimodal psychosomatic therapy in patients with medically unexplained symptoms: feasibility and treatment effects. Fam Pract.

[21] Wileman L, May C, Chew-Graham CA (2002). Medically unexplained symptoms and the problem of power in the primary care consultation: a qualitative study. Fam Pract.

[22] Reid S, Whooley D, Crayford T, Hotopf M (2001). Medically unexplained symptoms—GPs’ attitudes towards their cause and management. Fam Pract.

[23] Peters S, Stanley IAN, Rose M, Salmon P (1998). Patients with medically unexplained symptoms: sources of patients’ authority and implications for demands on medical care. Soc Sci Med..

[24] Grandes G, Montoya I, Arietaleanizbeaskoa M, Arce V, Sanchez A (2011). The burden of mental disorders in primary care. Eur Psychiatry.

[25] Cape J, Barker C, Buszewicz M, Pistrang N (2000). General practitioner psychological management of common emotional problems (I): Definitions and literature review. Br J Gen Pract.

[26] Robbins J, Kirmayer L, Hemami S (1997). Latent variable models of functional somatic distress. J Nerv Ment Dis.

[27] Kroenke K, Spitzer RL, Williams JB (2002). The PHQ-15: validity of a new measure for evaluating the severity of somatic symptoms. Psychosom Med.

[28] Zonneveld LNL, van’t Spijker A, Passchier J, van Busschbach JJ, Duivenvoorden HJ (2009). The effectiveness of a training for patients with unexplained physical symptoms: protocol of a cognitive behavioral group training and randomized controlled trial. BMC Public Health..

[29] Van Rood Y, van Ravesteijn H, de Roos C, Spinhoven P, Speckens A. Protocol for diagnosis and treatment of patients with medically unexplained physical symptoms: the consequences model (in Dutch). Keijsers, van Minnen & Hoogduin (eds): Protocolized treatments for adults with psychological complaints 2 (in Dutch). Amsterdam: Boom; 2011. p. 15–47.

[30] Speckens A, van Hemert A, Spinhoven P, Hawton KE, Bolk JH, Rooijmans HG (1995). Cognitive behavioural therapy for medically unexplained physical symptoms: a randomised controlled trial. Br Med J.

[31] Zonneveld LNL, Duivenvoorden HJ, Passchier J, Van Spijker A (2010). Tailoring a cognitive behavioural model for unexplained physical symptoms to patient’s perspective: a bottom-up approach. Clin Psychol Psychother.

[32] Mynors-Wallis LM, Gath DH, Day A, Baker F (2000). Randomised controlled trial of problem solving treatment, antidepressant medication, and combined treatment for major depression in primary care. Br Med J..

[33] Mynors-Wallis L, Gath D, Lloyd-Thomas A, Tomlinson D, Gath D, Tomlinson D (1995). Randomised controlled trial comparing problem solving treatment with amitriptyline and placebo for major depression in primary care. BMJ.

[34] Hassink-Franke LJA, van Weel-Baumgarten EM, Wierda E, Engelen M, Beek M, Bor H (2011). Effectiveness of problem-solving treatment by general practice registrars for patients with emotional symptoms. J Prim Health Care.

[35] Wilkinson P, Mynors-Wallis L (1994). Problem-solving therapy in the treatment of unexplained physical symptoms in primary care: a preliminary study. J Psychosom Res.

[36] Olde Hartman T, Blankenstein A, Molenaar A, Bentz van den Berg D, van der Horst H, Arnold I (2013). NHG guideline on medically unexplained symptoms (MUS) (in Dutch). Huisarts Wet.

[37] Hays RD, Morales LS (2001). The RAND-36 measure of health-related quality of life. Ann Med..

[38] Van der Zee K, Sanderman R, Heyink J, de Haes H (1996). Psychometric qualities of the RAND 36-item health survey 1.0: a multidimensional measure of general health status. Int J Behav Med.

[39] Van der Zee K, Sanderman R. Measuring general health with the RAND-36, a guideline (in Dutch). 2nd ed. Groningen: American Psychiatric Association; 2012.

[40] Herdman M, Gudex C, Lloyd A, Janssen M, Kind P, Parkin D (2011). Development and preliminary testing of the new five-level version of EQ-5D (EQ-5D-5 L). Qual Life Res.

[41] Versteegh MM, Vermeulen KM, Evers SMAA, De Wit GA, Prenger R, Stolk EA (2016). Dutch tariff for the five-level version of EQ-5D. Value Heal.

[42] Hakkaart-van Roijen L, van Straten A, Donker M, Tiemens B (2002). Manual Trimbos/IMTA questionnaire for costs associated with psychiatric illness (TIC-P).

[43] Zigmond A, Snaith R (1983). The Hospital Anxiety and Depression Scale. Acta Psychiatr Scand.

[44] American Psychiatric Association (2013). Diagnostic and statistical manual of mental disorders.

[45] Dreer LE, Berry J, Rivera P, Elliott TR, Miller D, Little TD (2009). Efficient assessment of social problem-solving abilities in medical and rehabilitation settings: a Rasch analysis of the Social Problem-Solving Inventory-Revised. J Clin Psychol.

[46] Pilowsky I (1967). Dimensions of hypochondriasis. Br J Psychiatry.

[47] Broadbent E, Petrie KJ, Main J, Weinman J (2006). The brief illness perception questionnaire. J Psychosom Res.

[48] Moss-Morris RE, Chalder T (2003). Illness representations: where to from here? Kos: 16th Conference of the European Health Psychology Society.

[49] Pearlin L, Schooler C (1978). The structure of coping. J Health Soc Behav.

[50] Stinckens N, Elliott R, Leijssen M (2009). Bridging the gap between therapy research and practice in a person-centered/experiential therapy training program: the Leuven systematic case study research protocol. Pers Exp Psychother..

[51] Samsa G, Edelman D, Rothman M, Willieams G, Lipscomb J, Matchar D (1999). Determining clinically important differences in health status measures: a general approach with illustration to the Health Utilities Index Mark II. Pharmacoeconomics..

[52] Farivar SS, Cunningham WE, Hays RD. Correlated physical and mental health summary scores for the SF-36 and SF-12 Health Survey, V.I. Health Qual Life Outcomes. 2007;5:54. 10.1186/1477-7525-5-54 PMC2065865 17825096

[53] Campbell MK, Fayers PM, Grimshaw JM (2005). Determinants of the intracluster correlation coefficient in cluster randomized trials: the case of implementation research. Clin Trials..

[54] Adams G, Gulliford MC, Ukoumunne OC, Eldridge S, Chinn S, Campbell MJ (2004). Patterns of intra-cluster correlation from primary care research to inform study design and analysis. J Clin Epidemiol.

[55] Campbell M, Piaggio G, Elbourne D, Altman D (2012). CONSORT 2010 Statement: extension to cluster randomised trials. BMJ..

[56] Fenwick E, O’Brien BJ, Briggs A (2004). Cost-effectiveness acceptability curves—facts, fallacies and frequently asked questions. Health Econ.

[57] Krull J, MacKinnon D (2016). Multilevel mediation modeling in group-based intervention studies. Eval Rev.

[58] Bell AC, D’Zurilla TJ (2009). Problem-solving therapy for depression: a meta-analysis. Clin Psychol Rev.

[59] Cuijpers P, van Straten A, Warmerdam L (2007). Problem solving therapies for depression: a meta-analysis. Eur Psychiatry.

[60] Frances A (2013). The new somatic symptom disorder in DSM-5 risks mislabeling many people as mentally ill. BMJ..

[61] Rief W, Martin A (2014). How to use the new DSM-5 somatic symptom disorder diagnosis in research and practice: a critical evaluation and a proposal for modifications. Annu Rev Clin Psychol..

[62] Katz J, Rosenbloom B, Fashler S (2015). Chronic pain, psychopathology, and DSM-5 somatic symptom disorder. Can J Psychiatry.

[63] Axelsson E, Andersson E, Ljótsson B, Wallhed Finn D, Hedman E (2016). The health preoccupation diagnostic interview: inter-rater reliability of a structured interview for diagnostic assessment of DSM-5 somatic symptom disorder and illness anxiety disorder. Cogn Behav Ther..

[64] Katsamanis M, Lehrer PM, Escobar JI, Gara MA, Kotay A, Liu R (2011). Psychophysiologic treatment for patients with medically unexplained symptoms: a randomized controlled trial. Psychosomatics.

[65] Gili M, Magallón R, López-Navarro E, Roca M, Moreno S, Bauzá N (2014). Health related quality of life changes in somatising patients after individual versus group cognitive behavioural therapy: a randomized clinical trial. J Psychosom Res.

[66] Sattel H, Lahmann C, Gündel H, Guthrie E, Kruse J, Noll-Hussong M (2012). Brief psychodynamic interpersonal psychotherapy for patients with multisomatoform disorder: randomised controlled trial. Br J Psychiatry.

[67] Schröder A, Rehfeld E, Ornbøl E, Sharpe M, Licht RW, Fink P (2012). Cognitive-behavioural group treatment for a range of functional somatic syndromes: randomised trial. Br J Psychiatry.

