# Supplementary material for: The CIPRUS study, a nurse-led psychological treatment for patients with undifferentiated somatoform disorder in primary care: study protocol for a randomised controlled trial
Source: Trials. 2017 May 3;18:206. doi: 10.1186/s13063-017-1951-2 (PMC5414236; doi:10.1186/s13063-017-1951-2)
Supplement: Supplementary file 1 — Schedule of enrolment, intervention and assessments. (DOC 39 kb) [file 13063_2017_1951_MOESM1_ESM.doc]

Additional file 1. Schedule of enrolment, interventions, and assessments.

|  | **STUDY PERIOD** | | | | | |
| --- | --- | --- | --- | --- | --- | --- |
|  | **Enrolment** | **Allocation** | **Post-allocation** | | | **Close-out** |
| **TIMEPOINT** | ***-1 month*** | ***0*** | ***2 months*** | ***4 months*** | ***8 months*** | ***12 months*** |
| **ENROLMENT:** |  |  |  |  |  |  |
| **Invitation by GP including screening questionnaire** | X |  |  |  |  |  |
| **Diagnostic interview** | X |  |  |  |  |  |
| **Informed consent** | X |  |  |  |  |  |
| **Allocation** |  | X |  |  |  |  |
| **INTERVENTIONS:** |  |  |  |  |  |  |
| **The nurse-led intervention** |  |  |  |  |  |  |
| **ASSESSMENTS:** | For overview of assessments see table 2. | | | | | |
|  |
