# Supplementary material for: The CIPRUS study, a nurse-led psychological treatment for patients with undifferentiated somatoform disorder in primary care: study protocol for a randomised controlled trial
Source: Trials. 2017 May 3;18:206. doi: 10.1186/s13063-017-1951-2 (PMC5414236; doi:10.1186/s13063-017-1951-2)
Supplement: Supplementary file 3 — List of participating primary care centres. (DOCX 15 kb) [file 13063_2017_1951_MOESM3_ESM.docx]

Additional file 3. Participating primary care centres

1. General practice Lindenzoom, Papendrecht, the Netherlands
2. General practice de Wilg, Papendrecht, the Netherlands
3. General practice Franssens & Wildeboer, Dordrecht, the Netherlands
4. General practice Oranjepark, Dordrecht, the Netherlands
5. Health care centre de Zorgmolen, Papendrecht, the Netherlands
6. General practice Keuning & Okma, Hendrik Ido Ambacht, the Netherlands
7. Health care centre Velserbroek, Velserbroek, the Netherlands
8. De Sluis medical centre, Spaarndam, the Netherlands
9. General practicioners Westerpark Bevers & Barten, Amsterdam, the Netherlands
10. General practitioners Dudokplein, Dordrecht, the Netherlands
11. Health care centre Marne, Amstelveen, the Netherlands
12. Medical centre de Steenpoort, Genemuiden, the Netherlands
13. General practice Hoofdweg 366, Amsterdam, the Netherlands
14. Health care centre Diemen Zuid, Diemen, the Netherlands
15. General practice de Kennemerpoort, Bennebroek, the Netherlands
16. General practice Roos, Laren, the Netherlands
17. General practice W.J. Vaarkamp, Badhoevedorp, the Netherlands
18. General practice Loosdrecht, Loosdrecht, the Netherlands
19. General practitioners Badhoevedorp, Badhoevedorp, the Netherlands
20. Health care centre Aletta Jacobsstraat, Apeldoorn, the Netherlands
21. OB/GYN general practice de Stethoscoop, Weesp, the Netherlands
22. General practice Bos, Weesp, the Netherlands
23. General practice A.C. Verhaar, Weesp, the Netherlands
24. Van Gaalen & Lesterhuis general practitioners, Weesp, the Netherlands
25. Medical Centre Pandion, Lelystad, the Netherlands
26. General practice ‘de Staart’, Dordrecht, the Netherlands
27. General practitioners Marknesse, Marknesse, the Netherlands
28. General practitioners Wijhe, Wijhe, the Netherlands
29. General practitioners Verhaar, Weesp, the Netherlands
30. General practice Casparus, Weesp, the Netherlands
31. General practice M.E. Toppers, Nieuw-Vennep, the Netherlands
32. General practice het Doktershuis, Ridderkerk, the Netherlands
33. General practice Maartensdijk, Maartensdijk, the Netherlands
34. General practice Nieuwe Tonge, Nieuwe Tonge, the Netherlands
35. General practice Den Bommel, Den Bommel, the Netherlands
36. General practice het Dok, Urk, the Netherlands
37. Health care centre Lunetten, Utrecht, the Netherlands
38. Health care centre Hoograven, Utrecht, the Netherlands
39. Medical centre de Pionier, Nieuw-Vennep, the Netherlands
